# Supplementary material for: Analysis of 16 studies in nine rodent models does not support the hypothesis that diabetic polyuria is a main reason of urinary bladder enlargement
Source: Front Physiol. 2022 Aug 8;13:923555. doi: 10.3389/fphys.2022.923555 (PMC9393211; doi:10.3389/fphys.2022.923555)
Supplement: Supplementary file 1 [file Table1.DOCX]

**Online Supplement to**

**Analysis of 16 studies in nine distinct rodent models does not support the hypothesis**

**that diabetic polyuria is a main reason of urinary bladder enlargement**

Zeynep E. Yesilyurt, Jan Matthes, Edith Hintermann, Tamara R. Castañeda, Ralf Elvert, Jesus H. Beltran-Ornelas, Diana L. Silva-Velasco, Ning Xia, Aimo Kannt, Urs Christen, David Centurion, Huige Li, Andrea Pautz, Ebru Arioglu-Inan, Martin C. Michel

All animals and data have been obtained from ongoing studies primarily performed for other purposes in the labs of the investigators. All studies were in line with applicable rules and regulations including NIH guidelines for the use of experimental animals and Directive 2010/63/EU of the European Parliament on the protection of animals used for scientific purposes. Data related to the primary purposes of those studies will be reported elsewhere. This Online Supplement includes the following information for each of the 16 studies:

- A description of each animal model
- Information on approval by the applicable animal committee
- A graphical depiction of values for each animal within each study
- A graphical depiction of correlations between glucose and insulin levels on the one and bladder and bladder/body weight data within each study. Outcomes of correlation analyses are shown as r^2^ values to indicate strength of correlation, irrespective whether the correlations had descriptive p-values < or ≥ 0.05.

# Data quality measures

Except for studies performed in Ankara and Hoechst, the underlying studies did not involve randomization and/or blinding based on their specific needs. The decision to collect data from a given study for the present analyses was made before any animal was sacrificed. We operationally defined normoglycemia as blood glucose concentrations of <8 mM, hyperglycemia as 8-16 mM, and overt diabetes as >16 mM.

Sample size for the present analyses had been specified as all available animals from a given study, and that decision had been made before data were viewed. All analyses had been specified prior to data collection with one exception: after one study had included insulin data (fructose-fed I), additional correlation analysis was done for that parameter; when this yielded a correlation strength of likely biological relevance, presence of insulin data was checked for each study and, where available, correlations of bladder phenotype with insulin levels were performed.

# Type 1 diabetes models

## STZ-injected rats (Mexico City)

The study had been approved by Institutional Ethics Committee (Cicual-Cinvestav; 0102-14). Male Wistar rats (about 250 g, 7 weeks old) were obtained from the laboratory animal facility of the Dept. of Pharmacobiology, Cinvestav, Sede Sur and housed in an air-conditioned room (temperature 22 ± 2 °C; 50% relative humidity) as part of a specific-pathogen-free facility under a 12/12 h light cycle (dark cycle from 18 h to 6 h). They were kept in acrylic cages measuring 43 x 53 x 20 cm with pine sawdust beds sterilized by UV light changed every 2 days and a maximum of 6 animals per cage.

Breeding program: The animals were reproduced by specialized technicians trained in the handling, use and reproduction of laboratory animals. Briefly, Wistar virgin female rats weighing approximately 200-220 g were placed in a 3:1 ratio with the male for mating. Mating took place in the heat stage and is influenced by the male's pheromones. The mating period lasted from 3 to 5 days. Subsequently, pregnant females were placed in individual acrylic cages (27X37X15 cm) for delivery and until weaning. The gestation period lasted from 22 to 23 days and an average of 12 pups per litter were obtained. The offspring was weaned on day 21 of lactation, where they are sexed and separated into collective boxes. Female rats were used a maximum of 3 times for reproduction.

In a 2-armed study, rats were injected i.p. with vehicle (citrate buffer pH 4.5) or 60 mg/kg streptozotocin (STZ). After 6 weeks, they were anesthetized with isoflurane (3%) and euthanized by decapitation.

Supplemental Figure 1: Blood glucose, body weight, bladder weight and bladder/body weight in STZ-injected rats. Each data point represents one animal, bars and error bars represent means ± SD.

Supplemental Figure 2: Correlation of blood glucose with bladder and bladder/body weight. Each data point represents one animal; the line represents the calculated regression line with its 95% CI. Descriptive p-values were 0.0261 and <0.0001, respectively.

## STZ-injected rats (Ankara)

The study had been approved by the animal welfare committee of Ankara University

(2019-4-41). In a 6-armed study, 11-week-old male Sprague Dawley rats were obtained from Bilkent University Genetics and Biotechnology Research Center and Kobay Experimental Animals Laboratory (Ankara, Turkey) and housed in the Animal Care Unit of Faculty of Pharmacy, Ankara University at a temperature of 20-24°C, humidity of 40-60% and a lighting cycle of 12:12 h in cages with a floor area of 42.5x26.6, height 18.5 cm and 3 animals per cage or a floor area: 44x34, height: 20 cm and 4 animals per cage.

Rats had free access to standard rat chow and drinking water. Rats were injected i.p. with vehicle or 50 mg/kg STZ; within each group, animals were further subdivided after 12-15 weeks by allocating them to treatment with vehicle, empagliflozin (30 mg/kg, with oral gavage) or linagliptin (4 mg/kg, with oral gavage). After 8-10 weeks of treatment, they were anesthetized under 2% isoflurane inhalation and sacrificed. Rats were randomized to group allocation and the person obtaining and weighing tissues was blinded to group allocation. Glucose levels had been measured in the week preceding sacrificing the rats.

Supplemental Figure 3: Blood glucose, body weight, bladder weight and bladder/body weight in STZ-injected rats with and without additional treatment with empagliflozin (empa) or linagliptin (lina). Each data point represents one animal, bars and error bars represent means ± SD. Note that measured glucose values were censored at 33.3 mM.

Supplemental Figure 4: Correlation of blood glucose with bladder and bladder/body weight. Each data point represents one animal; the line represents the calculated regression line with its 95% CI. Descriptive p-values were <0.0001 for both comparisons.

## Rat insulin promotor lymphocytic choriomeningitis virus (RIP-LCMV) mice (Frankfurt)

RIP-LCMV-GP mice express the glycoprotein (GP) of the lymphocytic choriomeningitis virus (LCMV) under control of the rat insulin promoter (RIP) in the beta-cells of the islets of Langerhans [1]. Infection of such mice with LCMV as environmental trigger initiates an immune response directed against LCMV as well as the transgenically expressed GP in the beta-cells resulting in type 1 diabetes within 10-14 days after infection [2]. Generation and screening by PCR of RIP-LCMV-GP transgenic mice were as previously described [1]. These mice have been backcrossed to a C57BL/6J background for more than 30 years. LCMV Armstrong clone 53b (LCMV-Arm) were plaque-purified three times on Vero cells and stocks were prepared by a single passage on BHK-21 cells [3].

The study had been approved by the Ethics Committee of the State Ministry of Agriculture, Nutrition and Forestry, State of Hessen, Germany (V54-19c20/15-FU-1192 and V54-19c20/15-FU-1213). Mice were bred in a specific pathogen free environment at mfd diagnostics (Wendelsheim, Germany) and tranferred for the experiments to our BSL-2 facility, where the mice are kept in isolated climate cabinets (Uni-Protect) in type 2 long cages (530 cm^2^) on spruce wood granulate bedding (Safe-Lab, Rosenberg, Germany) in litters of 3 to 5 mice. The mice were kept in isolated climate cabinets with a light/dark cycle of 12 hrs at a temperature of 20-24 °C and a humidity of 45-65%. The mice had access to food (complete maintainance diet for mice in coarse pellets (Safe A04); Safe-Lab, Rosenberg, Germany) ad libitum. Cages contained mouse igloos and nesting material as environmental enrichments. The health condition of the mice is evaluated accoding to a score sheet on a daily basis. Health monitoring is conducted on a regular basis for sentinal mice located in the same climate cabinet. In a 2-armed study, 8–10-week-old female and male RIP-LCMV-GP mice (C57BL/6J background) were obtained from mfd-diagnostics (Wendelsheim, Germany). Initial generation and screening by PCR of RIP-LCMV-GP mice (H-2^b^) were as previously described [3]. To induce type 1 diabetes, RIP-LCMV-GP mice were infected with 5 x 10^3^ plaque forming units (pfu; intraperitoneally in 100 µl RPMI medium). The majority (90-95%) of LCMV-infected RIP-LCMV-GP mice develop type 1 diabetes within 10-14 days after infection (blood glucose levels >16 mM). Blood glucose measurements were performed using a SD code-free blood glucose monitoring system (SD Biosensor, South Korea). Uninfected, age-matched female and male RIP-LCMV-GP mice were used as control. Mice were sacrificed 6-8 weeks after LCMV-infection by isoflurane overdose and cervical dislocation. For the current experiments, bladders have been removed post-mortem from LCMV-infected, but otherwise untreated and from control mice involved in other projects. Bladders have been homogenized in 3 ml TriReagent (Sigma-Aldrich, St. Louis, MO), and stored at -80C until further use.

Supplemental Figure 5: Blood glucose, body weight, bladder weight and bladder/body weight in RIP-LCMV mice. Each data point represents one animal, bars and error bars represent means ± SD. Note that measured glucose values were censored at 33.3 mM.

Supplemental Figure 6: Correlation of blood glucose with bladder and bladder/body weight in RIP-LMCV mice. Each data point represents one animal; the line represents the calculated regression line with its 95% CI. Descriptive p-values were <0.0001 in both cases.

# Type 2 diabetes models

## ZSF1 rats (Hoechst)

Two studies of similar design but with different treatment groups were performed in 7-8-week-old male ZSF1 rats (Charles River, Kingston, NY, USA). The first had a duration of 12 weeks (20 weeks old rats at time of sacrifice) and the second of 20 weeks (28 weeks old rats at time of sacrifice). Both studies had been approved by the Ethics Committee of the State Ministry of Agriculture, Nutrition and Forestry, State of Hessen, Germany (the V54 - 19 c 20/15-FH/Anz. 1012). Rats were randomized by body weight before allocation to the different diets (ssniff Spezialdiäten, GmbH, Soest, Germany). The 12-week study included 6 arms: lean ZSF1 control rats, obese ZSF1 rats, obese ZSF1 rats on AMLN diet [4], obese ZSF1 rats on an AMLN diet in which 15% primex had been replaced with canoletta, and obese ZSF1 rats on a 0% choline/0.2% methionine diet. The 20-week study had 8 arms: lean ZSF1 control rats, obese ZSF1 rats, obese ZSF1 rats on AMLN diet + 99.5% methylcellulose and 0.5% Tween-80 (vehicle for elafibranor), obese ZSF1 rats on AMLN diet + the PPAR-α/δ agonist elafibranor (30 mg/kg) [5], obese ZSF1 rats on 0% choline/0.2% methionine diet, obese ZSF1 rats on canoletta diet, obese ZSF1 rats on AMLN diet + oil (vehicle for CCl_4_), and obese ZSF1 rats on AMLN diet + CCl_4_ (0.2 mg/kg); of note, this dose of CCl_4_ mistakenly was much lower than planned and not considered to cause hepatic cirrhosis. Housing and husbandry were as described below for the HFD mice (Hoechst study), except that two rats were housed per cage and that cages where changes twice a week when polyuria occurred. At the end of the study the rats were euthanized by final exsanguination under isoflurane anaesthesia.

Supplemental Figure 7: Blood glucose, body weight, bladder weight and bladder/body weight in lean control and ZSF1 rats on standard diet or the indicated specific diets after 12 weeks. Each data point represents one animal, bars and error bars represent means ± SD.

Supplemental Figure 8: Correlation of blood glucose (upper panels) and insulin (lower panels) with bladder and bladder/body weight in in lean control and ZSF1 rats with or without specific diets after 12 weeks. Each data point represents one animal; the line represents the calculated regression line with its 95% CI. Descriptive p-values were 0.4461, 0.3329, 0.6192 and 0.1186, respectively. Note that insulin levels in all lean control rats were below detection limit (0.512 ng/l) and were entered at this value into the correlation analyses.

Supplemental Figure 9: Blood glucose, body weight, bladder weight and bladder/body weight in lean control and ZSF1 rats on standard diet or the indicated specific diets after 20 weeks. Each data point represents one animal, bars and error bars represent means ± SD.

Supplemental Figure 10: Correlation of blood glucose (upper panels) and insulin (lower panels) with bladder and bladder/body weight in in lean control and ZSF1 rats with or without specific diets after 20 weeks. Each data point represents one animal; the line represents the calculated regression line with its 95% CI. Descriptive p-values were <0.0001, <0.0001, 0.6192 and 0.1186, respectively. Note that insulin levels in 4 lean control rats were below detection limit (0.512 ng/ml) and were entered at this value into the correlation analyses.

## Fructose-fed rats (Mexico City)

Three studies of similar design but different observation periods were performed in fructose-fed rats [6]. All three studies had been approved by the institutional ethics committee (Cicual-Cinvestav, approval 0102-14). For housing and husbandry, see description on STZ-injected rats (Mexico City). In 2-armed studies, male Wistar rats (200-220 g, 7 weeks old) were obtained from the laboratory animal facility of the Dept. of Pharmacobiology, Cinvestav Sede Sur and fed a standard diet or a diet supplemented with a 15% fructose solution. After 16 weeks (study II) or 20 weeks (studies I and III), they were anesthetized with isoflurane (3%) and euthanized by decapitation.

Supplemental Figure 11: Blood glucose, body weight, bladder weight and bladder/body weight in study I of control and fructose-fed rats. Each data point represents one animal, bars and error bars represent means ± SD.

Supplemental Figure 12: Correlation of blood glucose (upper panels) or insulin (lower panels) with bladder and bladder/body weight in study I of control and fructose-fed rats. Each data point represents one animal; the line represents the calculated regression line with its 95% CI. Descriptive p-values were 0.7465, 0.8384, 0.0088 and 0.0129, respectively.

Supplemental Figure 13: Blood glucose, body weight, bladder weight and bladder/body weight in study II of control and fructose-fed rats. Each data point represents one animal, bars and error bars represent means ± SD.

Supplemental Figure 14: Correlation of blood glucose with bladder and bladder/body weight in study II of control and fructose-fed rats. Each data point represents one animal; the line represents the calculated regression line with its 95% CI. Descriptive p-values were 0.1473 and 0.0944, respectively.

Supplemental Figure 15: Blood glucose, body weight, bladder weight and bladder/body weight in study III of control and fructose-fed rats. Each data point represents one animal, bars and error bars represent means ± SD.

Supplemental Figure 16: Correlation of blood glucose and insulin with bladder and bladder/body weight in study III of control and fructose-fed rats. Each data point represents one animal; the line represents the calculated regression line with its 95% CI. Descriptive p-values were 0.4481, 0.4590, 0.7605 and 0.1529, respectively.

## Rats with neonatal STZ injection (Mexico City)

The study had been approved by the institutional ethics committee (Cicual-Cinvestav, approval 0102-14). For housing and husbandry, see description on STZ-injected rats (Mexico City). This was a 2-armed study: Male Wistar new-born rats (7-10 g, 3-5 days old) were obtained from the laboratory animal facility of the Dept. of Pharmacobiology, Cinvestav, Sede Sur and injected i.p. with vehicle (citrate buffer pH 4.5) or 70 mg/kg STZ. After 16 weeks, they were anesthetized with isoflurane (3%) and euthanized by decapitation.

Supplemental Figure 17: Blood glucose, body weight, bladder weight and bladder/body weight in control animals and rats with neonatal STZ injection. Each data point represents one animal, bars and error bars represent means ± SD.

Supplemental Figure 18: Correlation of blood glucose with bladder and bladder/body weight in control animals and rats with neonatal STZ injection. Each data point represents one animal; the line represents the calculated regression line with its 95% CI. Descriptive p-values were 0.0199 and 0.0003, respectively.

## IRS2 knock-out mice (Cologne)

Animal breeding, maintenance and experiments had been approved by the responsible federal state authority (Landesamt für Natur-, Umwelt- und Verbraucherschutz Nordrhein-Westfalen; 84-02.04.2016.A049 and 84-02.04.2016.A422). Breeding, husbandry and use of the animals complied with the guidelines from Directive 2010/63/EU of the European Parliament on the protection of animals used for scientific purposes. Up to five mice each were kept in individually ventilated cages (type II long) in a facility with restricted access. On a regular basis, sample mice from the animal facility were tested for specific pathogens. The mating was 1:1, usually with heterozygous animals. Breeding and maintenance were performed with a 12h/12h dark/light cycle and food and water ad libitum. No assessments or investigations were carried out until the animals were killed. This was a 2-armed study using mice from both sexes: Details of the IRS2 knock-out mouse model have been described [7, 8]. Tail or ear clips from 3-week-old mice were processed for genotyping. Mice had an C57BL/6J background and were kept in individually ventilated cages with a 12h/12h dark/light cycle and food and water *ad libitum*. We used rat/mouse maintenance food (V1554-703, ssniff Spezialitäten GmbH, Soest, Germany). Immediately after killing by cervical dislocation, urinary bladder was excised and weighed and blood glucose was measured using a blood-glucose meter (Accu-Check® Aviva, Roche Diagnostics Deutschland GmbH, Mannheim, Germany) with a drop of blood leaking from the cut tail.

Supplemental Figure 19: Blood glucose, body weight, bladder weight and bladder/body weight in control (C57BL/6) and IRS2 knock-out mice. Each data point represents one animal, bars and error bars represent means ± SD.

Supplemental Figure 20: Correlation of blood glucose with bladder and bladder/body weight in control (C57BL/6) and IRS2 knock-out mice. As the study had used mice aged 16-61 weeks, additional correlation analysis of age vs. bladder weight was performed to explore whether age differences may have affected outcomes; this has not been the case (lower panel). Each data point represents one animal; the line represents the calculated regression line with its 95% CI. Descriptive p-values were 0.0893, 0.1305 and 0.6776, respectively.

## ob/ob mice (Cologne)

This study used control (C57BL/6J background) and ob/ob mice (leptin B6.Cg-Lep ob/J) from both sexes initially purchased from the Jackson Laboratory (Bar Harbor, USA) and maintained at our facility. Ethical approval, husbandry, housing, breeding, genotyping and study conduct were identical with those described above for the IRS2 knock-out experiments.

Supplemental Figure 21: Blood glucose, body weight, bladder weight and bladder/body weight in control (C57BL/6) and ob/ob mice. Each data point represents one animal, bars and error bars represent means ± SD.

Supplemental Figure 22: Correlation of blood glucose with bladder and bladder/body weight in control (C57BL/6) and ob/ob mice. As the study had used mice aged 21-56 weeks, additional correlation analysis of age vs. bladder weight was performed to explore whether age differences may have affected outcomes; this has not been the case (lower panel). Each data point represents one animal; the line represents the calculated regression line with its 95% CI. Descriptive p-values were 0.7410, 0.9593 and 0.4126, respectively.

## ob/ob and db/db mice (Hoechst)

This was a study in which ob/ob and db/db mice were studied in comparison to wild-type C57BL/6J mice. As the underlying study involved only tissue harvesting and no experimentation or other intervention in living animals, no animal permit from the authorities was required according to §4 Abs. 3 of the German animal protection law (Tierschutzgesetz). Instead an internal permit for animal numbers to be reported (Tiertötung einer Maus, T4-12.A) was obtained. Mice of both sexes were obtained from Charles River Laboratories Germany GmbH (Sulzfeld, Germany) for tissue collection at an age of 12 weeks. Housing and husbandry were as described below for HFD mice (Hoechst). All mice were euthanized with an isoflurane overdose and euthanized by cervical dislocation, half within each group at 7 a.m. (fed stage) and half at 2 p.m. (starved stage). The graphs show pooled data of both sexes and both time points of euthanization.

Supplemental Figure 23: Blood glucose, body weight, bladder weight and bladder/body weight in control (C57BL/6J), db/db and ob/ob mice. All mice were euthanized with an isoflurane overdose and euthanized by cervical dislocation, half within each group at 7 a.m. (fed stage) and half at 2 p.m. (starved stage). The graphs show pooled data of both sexes and both time points of euthanization. Each data point represents one animal, bars and error bars represent means ± SD.

Supplemental Figure 24: Correlation of blood glucose with bladder and bladder/body weight in control (C57BL/6J), db/db and ob/ob mice. Each data point represents one animal; the line represents the calculated regression line with its 95% CI. Note that almost all animals in the upper left quarter of the glucose vs. bladder/body weight panel represent the control group driven by the lower body weight in absence of change in bladder weight in this group. Descriptive p-values were 0.2521 and <0.0001, respectively.

## HFD mice (Hoechst)

This was a 2-armed study in 12-week-old C57BL/6N control and 24-week-old HFD mice, which was performed in conjunction with the above study on db/db and ob/ob mice and had been approved by the Ethics Committee of the State Ministry of Agriculture, Nutrition and Forestry, State of Hessen, Germany (the V54 - 19 c 20/15-FH/Anz. 1024 and T4-12.A1). Mice of both sexes were obtained from Charles River Laboratories Germany GmbH (Sulfeld, Germany); the HFD group received an HFD (EF adjusted fat diet, 25% fat content; ssniff Spezialdiäten, GmbH, Soest, Germany) for 18 weeks for tissue collection at an age of 24 weeks. All mice were housed in a specific pathogen free like animal facility with increased hygienic status. While female mice were housed in groups with n=8 per cage and feeding stage (type Makrolon 4), the male mice were single housed (type Makrolon 2) to avoid fighting and serious injuries, with n=8 per group or feeding stage. The light/dark cycle was kept at 12:12h (dark period 18.00 to 6.00 h) at a room temperature of 23±1 °C and a relative humidity of 40-50%. All mice had free access to food and water or stated accordingly. The bedding material consisted of wooden crinklets (Rosenberg, Germany). The cages were enriched with sizzle nests, wooden sticks (both Rosenberg, Germany) for biting and mouse igloos as shelters (Plexx, Netherlands). The mice were checked daily for health status and were under continuous supervision of local veterinarians and animal welfare officer during pre-feeding on high-fat diet or, as the second HFD study below, during treatment period. No welfare-related interventions were monitored. All mice were euthanized with an isoflurane overdose and euthanized by cervical dislocation, half within each group at 7 a.m. (fed stage) and half at 2 p.m. (“fasted” stage). The graphs show pooled data of both sexes and both time points of euthanization.

Supplemental Figure 25: Blood glucose, body weight, bladder weight and bladder/body weight in mice of both sexes (C57BL/6N) on a control or an HFD. All mice were euthanized with an isoflurane overdose and euthanized by cervical dislocation, half within each group at 7 a.m. (fed stage) and half at 2 p.m. (“fasted” stage). Each data point represents one animal, bars and error bars represent means ± SD.

Supplemental Figure 26: Correlation of blood glucose with bladder and bladder/body weight in mice (C57BL/6N) on a control or HFD. Each data point represents one animal; the line represents the calculated regression line with its 95% CI. Descriptive p-values were 0.5655 and 0.1214, respectively.

## HFD mice + semaglutide treatment (Hoechst)

The study had been approved by the Ethics Committee of the State Ministry of Agriculture, Nutrition and Forestry, State of Hessen, Germany (the V54 - 19 c 20/15-FH/Anz. 1024). It was a 3-armed study: Male C67BL/6N mice (approximately 18-20 g body weight and 4-6 weeks of age) were obtained from Charles River (Sulzfeld, Germany) and placed on a control or an HFD for 18 weeks (EF adjusted fat diet, 25% fat content; ssniff Spezialdiäten, GmbH, Soest, Germany); thereafter, some of the mice with HFD received semaglutide [9] at a dose of 10 nmol/kg, every second day for another 36 days, i.e. approximately 29 weeks old at time of euthanization. Housing and husbandry were as described for the HFD mice Hoechst study. One mouse was excluded from the study after a glucose tolerance test on day 33 of treatment; no other interventions were monitored. Allocation to the three groups was based on randomization according to pre-treatment body weight. At study end, they were sacrificed by terminal bleeding from Vena cava caudalis under deep isoflurane anaesthesia.

Supplemental Figure 27: Blood glucose, body weight, bladder weight and bladder/body weight in male mice (C57BL/6N) on a control (n = 8), HFD (n = 8) and HFD + semaglutide (n = 7). Each data point represents one animal, bars and error bars represent means ± SD.

Supplemental Figure 28: Correlation of blood glucose with bladder and bladder/body weight in mice (C57BL/6JN) on a control or HFD. Each data point represents one animal; the line represents the calculated regression line with its 95% CI. Descriptive p-values were 0.1947, 0.2945, 0.2912 and 0.1322, respectively.

## HFD mice (Mainz)

The study was approved by the responsible regulatory authority (Landesunter­suchungs­amt Rheinland-Pfalz; 23 177-07/G 17-1-020). Male C57BL/6J mice were from Janvier Labs (Le Genest-Saint-Isle, France). The mice were kept in social groups of 3-5 per group in type II cages. The standard environment consisted of 250 g of bedding material (Midi, ABEDD, Vienna, Austria), one tunnel (Thyssenkrupp, Essen, Germany; PVC 100 mm x 40 mm) and one tissue paper (2 g, Green Singlefold Hand Towel Advanced, Tork, Dunstable UK). The mice were maintained under a 12:12-h light/dark cycle (lights on 06:00–18:00) in a temperature- and humidity-controlled animal room (22 ± 2 °C, 55 ± 5%). Mice were fed ad libitum either with normal control or HFD for 21 weeks, beginning at the age of 6 weeks. The HFD (E15744-34, corresponding to Research Diets D12451) was obtained from ssniff Spezialdiäten GmbH (Soest, Germany) and was a defined, lard-based diet with 45% energy from fat, 35% from carbohydrates and 20% from protein. Twice a week food was exchanged, and food consumption was assessed. Mice were housed in cages with three to five mice.

Supplemental Figure 29: Fasting blood glucose, body weight, bladder weight and bladder/body weight in mice (C57BL/6J) on a control or HFD. Each data point represents one animal, bars and error bars represent means ± SD.

Supplemental Figure 30: Correlation of blood glucose and insulin with bladder and bladder/body weight in control (C57BL/6J) and HFD mice. Each data point represents one animal; the line represents the calculated regression line with its 95% CI. Note that the correlation between glucose level and bladder/body weight is primarily driven by the body weight gain on an HFD. Descriptive p-values were 0.4783, 0.019, 0.2459 and 0.0470, respectively.

# References

[1] Oldstone MBA, Nerenberg M, Southern P, Price J, Lewicki H (1991) Virus infection triggers insulin-dependent diabetes mellitus in a transgenic model: role of anti-self (virus) immune response. Cell 65(2): 319-331. 10.1016/0092-8674(91)90165-U

[2] Christen U, Hintermann E, Holdener M, Von Herrath MG (2010) Viral triggers for autoimmunity: Is the ‘glass of molecular mimicry’ half full or half empty? J Autoimmun 34(1): 38-44. 10.1016/j.jaut.2009.08.001

[3] von Herrath MG, Dockter J, Oldstone MBA (1994) How virus induces a rapid or slow onset insulin-dependent diabetes mellitus in a transgenic model. Immunity 1(3): 231-242. 10.1016/1074-7613(94)90101-5

[4] Clapper JR, Hendricks MD, Gu G, et al. (2013) Diet-induced mouse model of fatty liver disease and nonalcoholic steatohepatitis reflecting clinical disease progression and methods of assessment. American Journal of Physiology-Gastrointestinal and Liver Physiology 305(7): G483-G495. 10.1152/ajpgi.00079.2013

[5] Su Z, Widomski D, Ma J, et al. (2016) Longitudinal changes in measured glomerular filtration rate, renal fibrosis and biomarkers in a rat model of type 2 diabetic nephropathy. Am J Nephrol 44(5): 339-353. 10.1159/000449324

[6] Tran LT, Yuen VG, McNeill JH (2009) The fructose-fed rat: a review on the mechanisms of fructose-induced insulin resistance and hypertension. Mol Cell Biochem 332(1): 145-159. 10.1007/s11010-009-0184-4

[7] Oliveira JM, Rebuffat SA, Gasa R, Gomis R (2014) Targeting type 2 diabetes: lessons from a knockout model of insulin receptor substrate 2. Can J Physiol Pharmacol 92(8): 613-620. 10.1139/cjpp-2014-0114

[8] Withers DJ, Gutierrez JS, Towery H, et al. (1998) Disruption of IRS-2 causes type 2 diabetes in mice. Nature 391(6670): 900-904. 10.1038/36116

[9] Lau J, Bloch P, Schäffer L, et al. (2015) Discovery of the once-weekly glucagon-like peptide-1 (GLP-1) analogue semaglutide. J Med Chem 58(18): 7370-7380. 10.1021/acs.jmedchem.5b00726
