# Supplementary material for: Analysis of 16 studies in nine rodent models does not support the hypothesis that diabetic polyuria is a main reason of urinary bladder enlargement
Source: Front Physiol. 2022 Aug 8;13:923555. doi: 10.3389/fphys.2022.923555 (PMC9393211; doi:10.3389/fphys.2022.923555)
Supplement: Supplementary file 2 [file Table2.DOCX]

Analysis of 16 studies in nine rodent models does not support the hypothesis that diabetic polyuria is a main reason of urinary bladder enlargement

**Zeynep E. Yesilyurt^1^, Jan Matthes^2^, Edith Hintermann^3^, Tamara R. Castañeda^4^, Ralf Elvert^4*^, Jesus H. Beltran-Ornelas^6^,** **Diana L. Silva-Velasco^6^, Ning Xia^7^, Aimo Kannt^4,5^, Urs Christen^3^, David Centurion^6^, Huige Li^7^, Andrea Pautz^7^, Ebru Arioglu-Inan^1^, Martin C. Michel^7*^**

^1^Dept. of Pharmacology, School of Pharmacy, Ankara University, Ankara, Turkey

^2^Dept. of Pharmacology, University of Cologne, Cologne, Germany

^3^Pharmazentrum, Goethe University, Frankfurt, Germany

^4^Sanofi Research and Development, Frankfurt, Germany

^5^Fraunhofer Institute for Translational Medicine and Pharmacology ITMP, Frankfurt, Germany

^6^Dept. of Pharmacobiology, Cinvestav IPN, Mexico City, Mexico

^7^Dept. of Pharmacology, Johannes Gutenberg University, Mainz, Germany

*: present affiliation: Evotec International GmbH, Göttingen, Germany

*** Correspondence:**Martin C. Michel
marmiche@uni-mainz.de

Keywords: animal model; bladder; diabetes; diet; glucosuria; hypertrophy; insulin; treatment

Abstract

The urinary bladder is markedly enlarged in the type 1 diabetes mellitus model of streptozotocin-injected rats, which may contribute to the frequent diabetic uropathy. Much less data exists for models of type 2 diabetes. Diabetic polyuria has been proposed as the pathophysiological mechanism behind bladder enlargement. Therefore, we explored such a relationship across nine distinct rodent models of diabetes including seven models of type 2 diabetes/obesity by collecting data on bladder weight and blood glucose from 16 studies with 2-8 arms each; some studies included arms with various diets and/or pharmacological treatments. Data were analysed for bladder enlargement and for correlations between bladder weight on the one and glucose levels on the other hand. Our data confirm major bladder enlargement in streptozotocin rats and minor if any enlargement in fructose-fed rats, db/db mice and mice on a high-fat diet; enlargement was present in some of five not reported previously models. Bladder weight was correlated with blood glucose as a proxy for diabetic polyuria within some but not other models, but correlations were moderate to weak except for RIP-LMCV mice (r^2^ of pooled data from all studies 0.0621). Insulin levels also failed to correlate to a meaningful extent. Various diets and medications (elafibranor, empagliflozin, linagliptin, semaglutide) had heterogeneous effects on bladder weight that often did not match their effects on glucose levels. We conclude that the presence and extent of bladder enlargement vary markedly across diabetes models, particularly type 2 diabetes models; our data do not support the idea that bladder enlargement is primarily driven by glucose levels/glucosuria.

# Introduction

Diabetes mellitus causes major morbidity and mortality related to cardiovascular, renal and ocular function [1]. Lower urinary tract dysfunction (LUTD) in general and that of the urinary bladder in particular are at least as common, occurring in 80% and 50% of diabetic patients, respectively [2]. While LUTD does not lead to major morbidity or mortality, it reduces the quality of life of the afflicted patients [3; 4] and their partners [5] by impairing social interactions during the day and sleep during the night; LUTD is also associated with emergency room visits, hospitalizations and loss of work productivity [6].

The pathophysiology of LUTD in diabetes is poorly understood and dedicated therapeutic strategies other than normalizing glucose levels are lacking. An enlargement of the urinary bladder appears to be part of LUTD in diabetes and is consistently found in the streptozotocin (STZ)-induced rat model of type 1 diabetes, by average resulting in a doubling of bladder weight (BW) [7]. While studied much less frequently, a comparable enlargement of the urinary bladder appears to exist in the other type 1 diabetes models that have been tested [8]. Much fewer studies have explored bladder enlargement in animal models of type 2 diabetes and have yielded inconsistent results [8]. Thus, it remains unclear whether bladder enlargement occurs in diabetes in general, is restricted to type 1 diabetes models or occurs in some but not all type 2 diabetes models. Treatment with insulin prevents and reverses bladder enlargement in STZ-injected rats [7]. However, no treatment studies have reported effects on BW in animal models of type 2 diabetes or with treatments other than insulin in those of type 1 diabetes.

The mechanisms underlying diabetes-associated bladder enlargement are largely unknown. A prevailing theory is that increased glucose levels act as an osmotic diuretic when exceeding the renal reabsorption threshold of 9-10 mM and that the bladder enlarges as a response to increased urine flow (diabetic polyuria). This theory is largely based on studies in rats in which treatment with the osmotic diuretic sucrose yielded similar degrees of diuresis and of bladder enlargement as compared to STZ injection [9; 10; 11; 12; 13; 14; 15]. It implies that bladder enlargement should be correlated to blood glucose levels if these exceed the renal reabsorption threshold. However, this mechanism has been questioned [8; 16].

Therefore, we have explored the presence and extent of bladder enlargement across a wide range of rodent models of diabetes, particularly of type 2 diabetes and including various diets and pharmacological treatments other than insulin and its correlation with blood glucose and, as a post-hoc analysis, serum insulin. For this purpose, we have collected data on glucose (in some cases also insulin), BW and body weight from various studies primarily designed to address questions unrelated to the urinary bladder. This has allowed us to collect data from 16 studies with 2-8 arms each representing nine distinct rat and mouse models and a total of 513 animals without sacrificing a single animal for the purpose of our study. Taken together we present what may be the most comprehensive inter-model comparison ever reported for any parameter in diabetes.

# Methods

## Animal models

To collect information from a wide range of rodent models of diabetes in the spirit of the 3R principles [17], the present study is based on data from ongoing studies designed for other purposes; primary outcomes of these studies will be reported elsewhere by the respective investigators. Details of each model according to the ARRIVE guidelines [18] are provided in the Online Supplement. Each of the underlying studies had been approved by the applicable independent committee or government agency for use and protection of experimental animals, and all studies were in line with the NIH guidelines for care and use of experimental animals (for details see Online Supplement). In each study, blood glucose concentration, body weight and BW were determined at study end in each animal and bladder/body weight ratio (BBW) was calculated. Plasma insulin levels were available from six studies. No treatments other than those being stated explicitly were applied.

## Data analysis

The following pre-specified analyses were done for each study: The primary outcome parameter within each study was BW, analysed as difference between the main hyperglycaemic/diabetic and its control group with its 95% CI as derived from an unpaired, two-tailed t-test assuming comparable variability in both groups. The key secondary outcome parameter within each study was the correlation between blood glucose and BW based on individual animal data of all groups with strengths of correlation assessed as the square of the Pearson correlation coefficient (r^2^) with its associated descriptive p-value. Other secondary outcome parameters were within-study group differences and correlations based on BBW. To explore correlations across groups, BW and BBW data from all animals other than those in the primary control group were expressed as % of the mean of the corresponding control group. This was followed by correlation analysis of the pooled data based on individual animal data across all models for comparison of BW and BBW vs. glucose. Similar correlations with insulin were done as post-hoc analyses.

In line with recent guidelines and recommendations [19; 20], we consider all analyses reported here as exploratory. Therefore, no hypothesis-testing statistical analysis was applied and reported p-values should be considered descriptive and not hypothesis-testing. We rather focus on reporting of effect sizes with their 95% confidence intervals (CI). All calculations were performed using Prism (v9.03; GraphPad, Los Angeles, CA, USA). Additional information on data quality measures is provided in the Online Supplement.

# Results

## Model characterization

### Glycaemic state

Based on an operational definition of normoglycaemia (<8 mM), hyperglycaemia (8-16 mM), and overt diabetes (>16 mM), some control groups were mildly hyperglycaemic (RIP-LMCV mice, one study each in C57BL/6J and in C57BL/6N mice). Similarly, these disease group did not exhibit overt diabetes in all studies (20-weeks old ZSF1 rats, rats with neonatal STZ injection, fructose-fed rats, ob/ob mice and mice on a high-fat diet (HFD)), and some diets and treatments (empagliflozin and semaglutide) lowered glucose in diabetic animals without restoring normoglycemia (Table 1).

<<<insert table 1 approximately here>>>

In all six studies with available insulin data, hyperinsulinemia relative to the respective control was observed (Table 1). Among treatments, canoletta and AMNL diets further increased insulin concentration in both ZSF1 rat studies, whereas 0% choline/0.2% methionine and elafibranor had no major effect; semaglutide lowered insulin concentration in HFD mice (Table 1).

### Body weight

Body weight was markedly reduced in STZ rats (>40%) and by <15% in RIP-LCMV mice (Table 1). Among type 2 diabetes/obesity models, body weight was markedly increased in ZSF1 rats of either age, in both studies with ob/ob mice, in db/db mice, and in HFD mice (Table 1). Fructose-feeding markedly increased body weight in two studies, but much less so in a third one (Table 1). Rats with neonatal STZ injection and IRS2 knock-out mice did not exhibit major alterations of body weight (Table 1). Empagliflozin and linagliptin had no major effects on body weight, whereas semaglutide normalized body weight and elafibranor reduced it by almost 40% relative to its control (AMNL vehicle; Table 1).

### Bladder enlargement

BW was increased in all type 1 diabetes models and in some type 2 diabetes/obesity models (both studies with ZSF1 rats, one of the three studies with fructose-fed rats, study with rats with neonatal STZ injection, both studies with ob/ob mice and in db/db mice; Table 1, Figure 1). In contrast, no bladder enlargement was observed in the other type 2 diabetes models (two out of three studies with fructose-fed rats, IRS2 knock-out mice, all three studies with HFD in mice). As body weight exhibited major changes in some of the models, a different picture was obtained for bladder/body weight (BBW; Table 1, Figure 1).

<<<insert figure 1 approximately here>>>

While HFD did not affect BW in mice (see above), addition of canoletta reduced BW in obese ZSF1 rats assessed at an age of 20 weeks (mean difference -56.7 mg [-86.2; -27.1]), a diet containing 0% choline/0.2% methionine increased BW (mean difference 75.0 mg [CI 8.6; 141.4]), and the AMLN diet had no detectable effect (mean difference -30 mg [CI -91.0; 31.0]; Table 1); however, all three estimates had wide CI making interpretation difficult. Similar effects of the three diets were seen at an age of 28 weeks.

Among pharmacological treatments, empagliflozin and linagliptin led to numerically large increases of BW in STZ rats, but these could not easily be interpreted due to large CI (mean difference 77.2 mg [CI -17.4; 171.8] and 81.6 mg [CI -11.3; 174.5], respectively; Table 1). Elafibranor induced a moderate reduction in BW as compared to obese ZSF1 rats on AMLN diet (mean difference -33 mg [CI -62.0; -4.0]). Semaglutide had no clear effect on BW (mean difference -6.7 mg [CI -18.4; 5.0]).

## Correlation analysis between blood glucose and bladder weight

Among models with blood glucose levels greater than the renal reabsorption threshold (>10 mM), IRS2 knock-out mice lacked and db/db exhibited only a minor increase in BW (Table 1, Figure 1). In contrast, bladder enlargement was observed in one study with a glucose level below the threshold (<9 mM; fructose-fed rat I), whereas studies with glucose levels approximately in the range of the threshold (9-10 mM) exhibited bladder enlargement in two but not in three other studies (Table 1, Figure 1).

Correlation analysis was performed within each model based on individual animal data (Table 2 and Figure 2). Strength of correlation between glucose level and BW (expressed as r^2^) varied markedly between models and ranged from 0.7226 in RIP-LCMV mice to 0.005 in one of the HFD mice studies. Except for the two ZSF1 rat studies, all groups had r^2^ values of <0.2, indicating that inter-animal variability of glucose levels, serving as a proxy of diabetic polyuria, statistically accounted for less than 20% of variability in BW. Comparable strength of correlation was found when glucose levels were compared to BBW; however, as a notable exception an r^2^ of 0.674 was found for db/db mice, a model in which BW was not markedly changed but body weight about doubled (Table 2). When data from the hyperglycaemic/diabetic animals of all studies were pooled, r^2^ was 0.0621 (Figure 2), indicating that glucose did not explain bladder weight variability in an inter-model analysis.

<<<insert table 2 approximately here>>>

<<<insert figure 2 approximately here>>>

## Correlation between serum insulin and bladder weight

In post-hoc correlation analyses between insulin levels and BW within each of the six studies with available insulin data (Table 3) and in a pooled analysis of all studies (Figure 3), a strong correlation was observed in one study with fructose-fed rats (r^2^ = 0.5127), this was neither confirmed in another study in this model nor in both studies with ZSF1 rats or in two studies with HFD; of note, a numerically inverse correlation was observed in one study with HFD mice (see Online Supplement). In a pooled analysis of data from all animals in the hyperglycaemic/diabetic groups, a week inverse correlation was observed (Figure 3, r^2^ = 0.0718, descriptive p-value 0.0077). Plasma insulin levels also positively correlated with BBW in the first fructose-feeding study but, if anything, inversely in the other five studies with available insulin data (Table 3).

<<<insert table 3 approximately here>>>

<<<insert figure 3 approximately here>>>

# Discussion

We have used data from 16 studies representing nine distinct rodent models of diabetes and 513 animals to address three specific questions:

- How widespread is urinary bladder enlargement in rodent models of experimental diabetes, particularly type 2 diabetes?
- How do diets and treatments other than insulin affect bladder enlargement?
- Is diabetic polyuria the key driver of diabetes-associated bladder enlargement across animal models?

## Critique of methods

It is a unique feature of the present study that it is fully based on data from experiments designed and conducted for other purposes. This is a limitation and a strength. The limitation results from the fact that the original studies were neither designed nor powered to explore bladder enlargement and its causes; moreover, the 16 studies were heterogeneous in species (rat and mouse), type 1 vs. type 2 diabetes, specific aspects of models including hereditary vs. acquired disease, duration of observation, and possible centre differences between contributing laboratories. To accommodate this limitation, we have expressed data in the hyperglycaemic/diabetic groups as % of the mean value in the corresponding euglycemic group for all inter-study analyses.

The 16 studies also varied in time from onset of diabetes to tissue harvesting, which raises the question whether that time period had been sufficient to induce the bladder weight phenotype. While none of the 16 studies had been designed to assess the bladder weight phenotype, we feel comfortable that time between onset and harvesting was sufficient to increase bladder weight if it occurs in a given model for two reasons. Firstly, each study had been designed and conducted to measure a specific phenotype; this target phenotype (distinct for each study) was reached in all studies. Second, we have previously analysed 83 groups of STZ vs. control rats [7]. Pooled analysis of extent of bladder enlargement vs. time suggested that bladder enlargement was largely complete after about 1 week after STZ injection. This was corroborated by looking at the time courses of the 10 studies that had tested 3 or more time points.

These limitations are outweighed by using an unprecedented number of models and studies. Given that each animal model of diabetes has limitations [21; 22], use of such variety of models should help to obtain data applicable to the heterogeneous population of diabetic patients [23]. Moreover, using data from studies designed for other purposes fulfils the ethical mandate of the 3R principles to reduce the use of experimental animals wherever possible [17]. Generating a comparable number of models and studies for the primary purpose of the present analyses would have been too resource-intensive to be justifiable and perhaps even unethical. Thus, the present analyses probably represent the largest collection of models and studies ever analysed for any outcome parameter within a single project in diabetes research.

## Bladder enlargement across models

More than 70 previous studies have demonstrated a consistent enlargement of the urinary bladder in rats injected with STZ (mean BW 178% of control; range 99-440%) [7]. A similar degree of enlargement was observed in a small number of studies with STZ-injected mice and rabbits, while other type 1 diabetes models including alloxan-injected rats and rabbits, BB/Wor rats and Akita mice exhibited a less pronounced increase in BW [8]. Our studies with STZ-injected rats (two reported here, a third reported elsewhere [24]) confirm these findings. Moreover, we extend this to another model of type 1 diabetes, RIP-LCMV mice, for which no BW data have been reported in the past.

Previous data in animal models of type 2 diabetes/obesity was limited to five models: fructose-fed rats, HFD mice, Goto-Kakizaki rats, Zucker diabetic fatty rats and db/db mice [8]. Across those models, bladder enlargement was small (about 150% of control) in fructose-fed rats and db/db mice, largely absent in HFD mice and in Goto-Kakizaki rats, but greater than the average enlargement in STZ-injected rats in Zucker diabetic fatty rats. Our present studies largely are in line with these findings. Our experiments also add data on four type 2 diabetes/obesity models for which bladder data had not been reported previously. We found a major increase in ZSF1 rats (>200% of control); as ZSF1 rats are a cross between Zucker diabetic fatty and spontaneously hypertensive rats and as Zucker rats were reported to exhibit a major bladder enlargement [8], these data are in line with previous findings. A moderate increase in bladder size was observed in rats injected with STZ at the neonatal stage and in ob/ob mice, whereas IRS2 knock-out mice did not exhibit bladder enlargement. In conclusion, the present data almost double the number of models of type 2 diabetes for which BW data have been reported. Together with data from previous systematic reviews [7; 8], these findings indicate that all animal models of type 1 diabetes exhibit bladder enlargement, although perhaps to a different extent, whereas BW increases markedly in some models of type 2 diabetes, only moderately in others and not at all in additional models. Apparently, severity of diabetes as assessed by blood glucose levels does not explain the observed heterogeneity of bladder enlargement. While the reasons for this heterogeneity are not fully clear, it is interesting that subgroups of patients with type 2 diabetes exhibiting distinct phenotypes are now also being recognized [23].

Other than in diabetes, bladder enlargement occurs in many conditions in animal models and patients, including bladder outlet obstruction and bladder denervation [25]. It typically is associated with LUTD. Therefore, a better understanding of the pathophysiology underlying diabetes-associated bladder enlargement may help to define innovative treatment strategies to combat frequent LUTD in diabetic patients.

## Differential effects of diets and pharmacological treatments

The present studies are the first to explore effects of drug treatments other than insulin [8] on diabetes-associated bladder enlargement. The four drugs applied in the underlying studies had the expected effects or lack thereof on glucose levels for the model in which they were used but, like the diets, did not affect glucose and BW in the same way in several cases: empagliflozin (a glycosuric drug [26]) lowered glucose but, if anything, increased BW; linagliptin (a drug not affecting glucosuria) tested within the same study caused a similar extent of bladder enlargement without affecting glucose levels. Semaglutide lowered glucose without affecting BW, and elafibranor did not affect glucose but reduced BW. These differential effects of diets and drug treatments are not easy to interpret because none of the studies had been designed to compare diet or drug effects on glucose and BW and because CI were wide in several cases. Nonetheless, the divergent effects casted doubt on the assumption that diabetic polyuria is the main reason for bladder enlargement.

## Role of glucose and insulin in bladder enlargement

When blood glucose levels exceed the renal reabsorption threshold, the excreted glucose can act as an osmotic diuretic and cause diabetes-associated polyuria. It had been proposed that such polyuria is the main cause for bladder enlargement in experimental diabetes. Support for this hypothesis largely comes from studies in which feeding with sucrose caused a similar degree of diuresis as STZ injection and a similar degree of bladder enlargement [9; 10; 11; 12; 13; 14; 15]. The polyuria hypothesis mechanistically implies that the degree of enlargement should be correlated with blood glucose levels because glucose levels determine the extent of diabetic polyuria. However, the presence of bladder enlargement segregated only poorly with glucose levels relative to the renal reabsorption threshold in our analyses of 16 studies.

To further test the diabetic polyuria hypothesis, we have previously correlated the reported glucose levels and bladder size alterations at the group level across a total of >100 studies: while we detected a correlation at the group level, it was only of moderate strength, i.e., less than 20% in variability of BW could mathematically be attributed to that of glucose levels [8]. A major limitation of that analysis was that we only had access to data at the group level. We performed a similar correlation analysis based on individual animal data for glucose level and BW in a recent pilot study, which also yielded a correlation of only moderate strength [24]. Therefore, individual animal-based correlation analyses were performed for the 16 studies reported here as a pre-specified outcome parameter (Table 2). BW was correlated with blood glucose concentration in the three studies with type 1 diabetes models but only in three out of 13 studies in type 2 diabetes/obesity models. Moreover, the strength of correlation varied markedly across models. Thus, a strong correlation was observed in RIP-LCMV mice, a moderate correlation in STZ-injected rats, ZSF1 rats and rats with neonatal STZ injection, but correlations were very weak if existing at all in the other models. To corroborate these findings, we also performed a correlation analysis based on pooled individual animals from the hyperglycaemic/diabetic groups of all 16 studies, which yielded an r^2^ of 0.0621 (Figure 2). While a positive correlation does not prove a cause-effect relationship, lack of correlations makes it unlikely that such relationship exists to a biologically meaningful extent. Taken together, these data do not support the hypothesis that polyuria is the main factor to explain diabetes-associated bladder enlargement.

Insulin is not only a hormone but also a growth factor [27], and fructose-fed rats often exhibit a greater increase in insulin than in glucose levels, possibly reflecting peripheral insulin resistance [28]. After having noticed a moderate to strong correlation of bladder enlargement with insulin levels in one study with fructose-fed rats (r^2^ = 0.5127), we performed a similar post-hoc analysis on the other five studies with available insulin data: all five studies including another study in fructose-fed rats exhibited very weak, and in one of them and in the pooled analysis of all studies numerically inverse correlations (Figure 3, Table 3). This is not too surprising given that type 1 diabetes is characterized by a reduced presence of insulin; while insulin can be increased in models of type 2 diabetes including those reported here, this effect typically is counterbalanced by a reduced insulin sensitivity.

Thus, our data on diets, drug treatments, blood glucose levels relative to the renal reabsorption threshold and most importantly our correlations between glucose and BW at the individual animal level do not support the diabetic polyuria hypothesis of bladder enlargement in animal models of type 2 diabetes. While this mechanism may play a role in some models such as RIP-LCMV mice, and perhaps a more moderate one in STZ-injected rats, it plays only a very minor if any role in most other models. More generally, our data suggest that animal models of diabetes not only differ in the presence and extent of bladder enlargement, but also in the pathophysiology leading to such enlargement in the models where it occurs. This conclusion is in line with the proposal that human type 2 diabetes is a heterogeneous condition with multiple underlying subgroups [23].

## Conclusions

Based on an unprecedented number of studies and animal models, we have shown that bladder enlargement is ubiquitous in animal models of type 1 diabetes and common, but not consistently present in those of type 2 diabetes/obesity. This heterogeneity among type 2 diabetes models is not explained by the severity of diabetes/hyperglycaemia, specifically not by glucose levels relative to the renal reabsorption threshold. For the first time, we have explored effects of various diets and drug treatments other than insulin on diabetes-associated bladder enlargement; many of them had differential effects on glucose levels and bladder enlargement. These differential effects together with the generally moderate to absent association of glucose levels with BW do not support the hypothesis that diabetic polyuria is the main cause of diabetes-associated bladder enlargements – at least in most models. Refuting the polyuria hypothesis generates the necessity for additional studies to identify alternative mechanisms leading to bladder enlargement in some experimental models of diabetes. Our analyses highlight the heterogeneity between animal models of diabetes. While type 2 diabetes patients apparently also are a heterogeneous group [23], specific links between such subgroups and specific animal models remain to be established. Finally, our data demonstrate that major research accomplishments can be made without use of extra animals if smart planning is applied.

# Conflict of Interest

TRC, RE and AK are former employees of Sanofi-Aventis. All other authors declare that the research was conducted in the absence of any commercial or financial relationships that could be construed as a potential conflict of interest.

# Author Contributions

ZEY: experimentation for STZ study Ankara; overall data analysis; co-development of primary manuscript draft; critical reading of manuscript for important intellectual content and approval of final manuscript.

JM: collection of data and supervision of study with ob/ob and IRS2 knock-out mice; critical reading of manuscript for important intellectual content and approval of final manuscript.

EH: experimentation for RIP-LCMV mouse study; critical reading of manuscript for important intellectual content and approval of final manuscript.

TRC: planning and experimentation for studies in ZSF1 rats and C57BL/6J, C57BL/6N, db/db, ob/ob and HFD mice; critical reading of manuscript for important intellectual content and approval of final manuscript.

RE: planning and experimentation in C57BL/6N and HFD mice; critical reading of manuscript for important intellectual content and approval of final manuscript.

JHBO: experimentation for fructose-fed and STZ rat studies; critical reading of manuscript for important intellectual content and approval of final manuscript.

DLSV: experimentation for neonatal STZ rat study; critical reading of manuscript for important intellectual content and approval of final manuscript.

NX: data collection and study supervision of the HFD model (Mainz); critical reading of manuscript for important intellectual content and approval of final manuscript.

AK: planning of studies in in ZSF1 rats and C57BL/6J, C57BL/6N, db/db, ob/ob and HFD mice, critical reading of manuscript for important intellectual content and approval of final manuscript.

UC: experimentation for RIP-LCMV mouse study; critical reading of manuscript for important intellectual content and approval of final manuscript.

DC: supervision of fructose-fed, STZ and neonatal STZ rat studies; critical reading of manuscript for important intellectual content and approval of final manuscript.

HL: study supervision of the HFD model (Mainz); critical reading of manuscript for important intellectual content and approval of final manuscript.

AP: conceptualization of overall project; critical reading of manuscript for important intellectual content and approval of final manuscript.

EAI: supervision of STZ study Ankara; co-lead of overall project; critical reading of manuscript for important intellectual content and approval of final manuscript.

MCM: conceptualization and lead of overall project; supervision of data analysis; co-development of primary manuscript draft; critical reading of manuscript for important intellectual content and approval of final manuscript.

# Funding

This work was funded in part by TÜBITAK 2211/A (to ZEY), TÜBITAK-SBAG 118S443 and 119S769 (to EAI), Landesoffensive zur Entwicklung wissenschaftlich-ökonomischer Exzellenz (LOEWE; LOEWE Center for Translational Medicine and Pharmacology) of the State of Hessen, Germany (to UC), Conacyt Mexico 252702 (to DC), and Deutsche Forschungsgemeinschaft XI 139/2-1 (to NX), LI-1042/5-1 (to HL) and Mi 294/10-1 (to MCM). Some underlying studies were performed and/or funded by Sanofi-Aventis (identified as “Hoechst”) for purposes unrelated to this manuscript. The commercial and non-commercial funders were not involved in the design of the study; the collection, analysis, and interpretation of data; writing the report; and did not impose any restrictions regarding the publication of the report.

# Tables

Table 1: Blood glucose, insulin (selected studies only), body weight, bladder weight, and bladder/body weight across animal models. Data are shown as means ± SD of the indicated number of animals. Insulin concentrations were below detection limit (0.000512 ng/ml) in lean ZSF1 rats in all animals in the 20- and 4/6 in the 28-week study; for calculation purposes they were set to 0.000512 ng/ml. Data from each individual animal of each study are shown in the Online Supplement.

|  | n | Blood glucose, mM | Insulin, ng/l | Body weight, g | Bladder weight, mg | Bladder/body weight, mg/g |
| --- | --- | --- | --- | --- | --- | --- |
| **type 1 diabetes models** | | | | | | |
| **STZ-injected rats (Mexico City)** | | | | | | |
| Control | 11 | 5.48±0.48 | - | 426.3±46.0 | 134.2±32.1 | 0.314±0.066 |
| STZ | 10 | 28.01±3.98 | - | 244.4±36.1 | 171.0±28.5 | 0.710±0.161 |
| **STZ-injected rats (Ankara)** | | | | | | |
| Control | 11 | 5.56±0.25 | - | 511.5±80.5 | 122.8±12.0 | 0.245±0.042 |
| Empagliflozin | 14 | 5.15±0.25 | - | 526.3±73.2 | 177.3±28.6 | 0.346±0.090 |
| Linagliptin | 12-13 | 5.57±0.38 | - | 532.5±86.2 | 158.7±53.5 | 0.307±0.103 |
| STZ | 13-14 | 31.31±3.91 | - | 327.0±78.6 | 291.7±41.9 | 0.900±0.267 |
| STZ+empagliflozin | 15 | 19.38±7.80 | - | 334.8±76.2 | 368.9±160.9 | 1.215±0.745 |
| STZ+linagliptin | 14 | 31.95±2.27 | - | 336.6±60.4 | 373.3±157.3 | 1.210±0.822 |
| **RIP-LCMV mice (Frankfurt)** | | | | | | |
| Control | 15 | 8.31±1.09 | - | 27.51±5.96 | 24.27± 5.02 | 0.891±0.115 |
| RIP-LCMV-GP | 12 | 28.34±8.49 | - | 24.04±3.72 | 43.00±14.21 | 1.830±0.696 |
| **type 2 diabetes models** | | | | | | |
| **ZSF1 rats (20-week, Hoechst)** | | | | | | |
| Lean control | 6 | 4.54±0.86 | <0.512 | 461.7±32.3 | 95.0±16.4 | 0.207±0.039 |
| Obese | 6 | 12.97±2.68 | 4.858±1.957 | 603.0±14.2 | 193.3±29.4 | 0.321±0.052 |
| Obese canoletta | 6 | 8.59±0.75 | 9.118±2.883 | 804.3±31.8 | 136.7±13.7 | 0.170±0.021 |
| Obese 0% choline/0.2% methionine | 6 | 13.87±4.47 | 5.313±1.571 | 819.3±31.7 | 268.3±66.8 | 0.328±0.080 |
| Obese AMLN | 6 | 8.58±1.62 | 8.100±3.405 | 798.2±26.8 | 163.3±60.2 | 0.206±0.077 |
| **ZSF1 rats (28-week, Hoechst)** | | | | | | |
| Lean control | 6 | 5.35±0.35 | 0.676±0.271 | 535.5±33.0 | 113.3±10.3 | 0.213±0.024 |
| Obese | 6 | 16.63±1.26 | 3.517±0.766 | 679.9±37.9 | 231.7±39.7 | 0.340±0.046 |
| Obese canoletta | 6 | 9.31±1.52 | 8.160±3.573 | 1082±58.5 | 156.7±10.3 | 0.145±0.00/ |
| Obese 0% choline/0.2% methionine | 5 | 14.94±2.68 | 5.542±1.744 | 795.7±26.5 | 378.0±151.7 | 0.474±0.186 |
| Obese AMLN-vehicle | 5 | 10.82±1.03 | 7.678±1.673 | 1029±31.8 | 156.0±37.8 | 0.152±0.041 |
| Obese AMLN- elafibranor (30 mg/kg) | 5 | 9.40±1.09 | 4.260±1.187 | 873.6±44.9 | 132.0 ± 8.4 | 0.152±0.016 |
| Obese AMLN-oil | 6 | 12.00±0.99 | 7.857±0.551 | 1072±73.1 | 165.0 ±27.4 | 0.155±0.030 |
| Obese AMLN- CCl_4_ (0.2 mg/kg) | 6 | 11.00±1.45 | 7.940±1.640 | 1065±76.3 | 155.0 ±33.9 | 0.146±0.034 |
| **Fructose-fed rats I (Mexico City)** | | | | | | |
| Control | 6 | 4.67±0.52 | 3.302±1.347 | 546.0±30.9 | 130.5 ±10.7 | 0.240±0.030 |
| Fructose-fed | 6 | 4.88±0.56 | 7.902±0.292 | 564.7±48.3 | 209.5 ±22.4 | 0.374±0.052 |
| **Fructose-fed rats II (Mexico City)** | | | | | | |
| Control | 6 | 4.67±0.52 | - | 468.3±42.2 | 146.5±10.3 | 0.315±0.034 |
| Fructose-fed | 6 | 4.95±0.76 | 5.938±2.572 | 535.7±48.2 | 136.1±29.5 | 0.254±0.048 |
| **Fructose-fed rats III (Mexico City)** | | | | | | |
| Control | 8 | 5.20±0.54 | 4.172±2.538 | 518.8±56.2 | 159.0±21.6 | 0.307±0.037 |
| Fructose-fed | 8 | 6.15±0.92 | 9.831±2.548 | 623.3±46.9 | 158.1±21.0 | 0.254±0.032 |
| **Rats with neonatal STZ injection (Mexico City)** | | | | | | |
| Control | 8 | 3.80±0.84 | - | 464.8±41.2 | 147.8±29.3 | 0.318±0.059 |
| Neonatal STZ | 8 | 9.06±7.03 | - | 420.8±58.0 | 184.6±39.3 | 0.453±0.149 |
| **IRS2 knock-out mice (Cologne)** | | | | | | |
| C57BL/6J | 12 | 8.98±1.59 | - | 31.52±5.70 | 30.28±6.11 | 0.982±0.217 |
| IRS2 knock-out | 12 | 16.02±9.27 | - | 31.73±5.27 | 25.96±7.30 | 0.824±0.230 |
| **ob/ob mice (Cologne)** | | | | | | |
| C57BL/6J | 9 | 9.40±2.24 | - | 30.19±5.32 | 25.89±5.64 | 0.865±0.175 |
| ob/ob | 14 | 9.19±2.91 | - | 64.70±6.09 | 36.59±13.05 | 0.565±0.195 |
| **ob/ob and db/db mice (Hoechst)** | | | | | | |
| C57BL/6J | 31 | 7.89±1.25 | - | 23.57±3.61 | 23.52±4.50 | 1.000±0.135 |
| ob/ob | 31 | 14.88±8.05 | - | 46.56±16.16 | 28.80±10.40 | 0.557±0.222 |
| db/db | 32 | 26.03±4.33 | - | 49.03±2.78 | 25.94±4.31 | 0.530±0.090 |
| **HFD mice (Hoechst)** | | | | | | |
| C57BL/6N | 32 | 7.64±0.95 | - | 23.97±2.81 | 28.25±5.93 | 1.177±0.201 |
| C57BL/6N HFD | 32 | 9.36±1.15 | - | 47.08±4.12 | 31.10±9.81 | 0.660±0.202 |
| **HFD mice + semaglutide (Hoechst)** | | | | | | |
| C67BL/6N | 8 | 9.35± 0.62 | 643.8±151.7 | 34.76±0.71 | 66.91±31.93 | 1.930±0.923 |
| C67BL/6N HFD | 8 | 9.23± 0.54 | 1021±263.2 | 43.72±2.66 | 44.25±12.44 | 1.025±0.345 |
| HFD+semaglutide | 7 | 7.92± 0.62 | 682.9±228.4 | 36.26±2.04 | 37.56± 7.43 | 1.045±0.253 |
| **HFD mice (Mainz)** | | | | | | |
| C57BL/6J | 12 | 5.96±0.72 | 284.8±205.5 | 34.07±2.65 | 33.33±5.33 | 0.980±0.149 |
| C57BL/6J HFD | 12 | 9.68±1.82 | 4431±819 | 49.25±2.31 | 35.17±6.93 | 0.713±0.125 |

Table 2: Correlation between blood glucose and bladder and bladder/body weight across animal models. Animals from diabetic and non-diabetic group were pooled for each correlation analysis. Shown are total number of animals per model, squared correlation coefficient (r^2^) and descriptive p-value. ^*^: negative slope (inverse correlation). A graphical representation of representative groups is shown in Figure 2, all other groups in the Online Supplement.

| n total | Bladder weight | | Bladder/body weight | |
| --- | --- | --- | --- | --- |
|  | r^2^ | p | r^2^ | p |
| **type 1 diabetes models** | | | | |
| **STZ-injected rats (Mexico City)** | | | | |
| 21 | 0.2346 | 0.0261 | 0.6368 | <0.0001 |
| **STZ-injected rats (Ankara)** | | | | |
| 79 | 0.3795 | <0.0001 | 0.3220 | <0.0001 |
| **RIP-LCMV mice (Frankfurt)** | | | | |
| 27 | 0.7226 | <0.0001 | 0.7322 | <0.0001 |
| **type 2 diabetes models** | | | | |
| **ZSF1 rats (20-week, Hoechst)** | | | | |
| 30 | 0.3632 | 0.0004 | 0.2428 | 0.0057 |
| **ZSF1 rats (28-week, Hoechst)** | | | | |
| 45 | 0.4127 | <0.0001 | 0.3168 | <0.0001 |
| **Fructose-fed rats I (Mexico City)** | | | | |
| 12 | 0.0109 | 0.7465 | 0.0044 | 0.8384 |
| **Fructose-fed rats II (Mexico City)** | | | | |
| 12 | 0.1979^*^ | 0.1473 | 0.2545^*^ | 0.0944 |
| **Fructose-fed rats III (Mexico City)** | | | | |
| 14 | 0.0488 | 0.4481 | 0.0465^*^ | 0.4590 |
| **Rats with neonatal STZ injection (Mexico City)** | | | | |
| 16 | 0.3302 | 0.0199 | 0.6262 | 0.0003 |
| **IRS2 knock-out mice (Cologne)** | | | | |
| 24 | 0.1256 | 0.0893 | 0.1009 | 0.1305 |
| **ob/ob mice (Cologne)** | | | | |
| 23 | 0.0053 | 0.7410 | 0.0001 | 0.9593 |
| **ob/ob mice (Hoechst)** | | | | |
| 62 | 0.0339 | 0.1519 | 0.0761^*^ | 0.0300 |
| **db/db mice (Hoechst)** | | | | |
| 63 | 0.1203 | 0.0054 | 0.6743* | <0.0001 |
| **HFD mice (Hoechst)** | | | | |
| 64 | 0.0054 | 0.5655 | 0.0383 | 0.1214 |
| **HFD mice + semaglutide (Hoechst)** | | | | |
| 23 | 0.0787 | 0.1947 | 0.0522 | 0.2945 |
| **HFD mice (Mainz)** | | | | |
| 24 | 0.0231 | 0.4783 | 0.3614* | 0.0019 |

Table 3: Correlation between plasma insulin and bladder and bladder/body weight across animal models of type 2 diabetes. Animals from diabetic and non-diabetic group were pooled for each correlation analysis. Shown are total number of animals per model, squared correlation coefficient (r^2^) and descriptive p-value. *: negative slope (inverse correlation). A graphical representation of representative groups is shown in Figure 3, all other groups in the Online Supplement.

| n total | Bladder weight | | Bladder/body weight | |
| --- | --- | --- | --- | --- |
|  | r^2^ | p | r^2^ | P |
| **ZSF1 rats (20-week, Hoechst)** | | | | |
| 30 | 0.0209 | 0.4461 | 0.0335^*^ | 0.3329 |
| **ZSF1 rats (28-week, Hoechst)** | | | | |
| 45 | 0.0058* | 0.6192 | 0.0557* | 0.1186 |
| **Fructose-fed rats I (Mexico City)** | | | | |
| 12 | 0.5127 | 0.0088 | 0.4773 | 0.0129 |
| **Fructose-fed rats III (Mexico City)** | | | | |
| 14 | 0.0080 | 0.7605 | 0.1626^*^ | 0.1529 |
| **HFD mice + semaglutide (Hoechst)** | | | | |
| 23 | 0.0529^*^ | 0.2912 | 0.1046^*^ | 0.1322 |
| **HFD mice (Mainz)** | | | | |
| 18 | 0.1319 | 0.2459 | 0.3389^*^ | 0.0470 |

# Legends to the figures

## Figure 1: Bladder and bladder/body weight differences across studies.

Data are shown as effect sizes comparing the primary hyperglycaemic/diabetic vs. the control group expressed as mean difference with its 95% confidence interval. Note that the same control group was used in the calculation of the ob/ob and db/db Hoechst groups.

## Figure 2: Correlation of bladder and bladder/body weight with glucose levels.

To enable pooling of data from all studies, those for the upper left panel shows bladder weight only from the non-control groups expressed as % of mean values in the control group within a study. The other three panels show correlations within three representative studies; data from the remaining studies are shown in the Online Supplement. A quantitative description of the correlations is shown in Table 2. Mean values of bladder weight and glucose level in each study are shown in Table 1.

## Figure 3: Correlation of bladder weight with insulin levels

To enable pooling of data from all studies, those for the upper left panel shows bladder weight only from the non-control groups expressed as % of mean values in the control group within a study. The other three panels show correlations within three representative studies; data from the remaining studies are shown in the Online Supplement. A quantitative description of the correlations is shown in Table 3. Mean values of bladder weight and insulin level in each study are shown in Table 1.

# References

[1] G.A. Mensah, G.S. Wei, P.D. Sorlie, L.J. Fine, Y. Rosenberg, P.G. Kaufmann, M.E. Mussolino, L.L. Hsu, E. Addou, M.M. Engelgau, and D. Gordon, Decline in cardiovascular mortality: possible causes and implications. Circulation Research 120 (2017) 366-380.

[2] F. Daneshgari, and C. Moore, Diabetic uropathy. Seminars in Nephrology 26 (2006) 182-185.

[3] J.S. Benner, R. Becker, K. Fanning, Z. Jumadilova, T. Bavendam, and L. Brubaker, Bother related to bladder control and health care seeking behavior in adults in the United States. Journal of Urology 181 (2009) 2591-2598.

[4] D.E. Irwin, I. Milsom, Z. Kopp, and P. Abrams, Symptom bother and health care-seeking behavior among individuals with overactive bladder. European Urology 53 (2008) 1029-1039.

[5] D. Mitroupoulos, I. Anastasiou, C. Giannopoulo, G. Nikolopoulos, C. Alamanis, and A. Zervas, Symptomatic benign prostatic hyperplasia: impact on partners' quality of life. European Urology 41 (2002) 240-245.

[6] H. Kannan, L. Radican, R.S. Turpin, and S.C. Bolge, Burden of illness associated with lower urinary tract symptoms including overactive bladder/urinary incontinence. Urology 74 (2009) 34-38.

[7] E. Arioglu Inan, J.H. Ellenbroek, and M.C. Michel, A systematic review of urinary bladder hypertrophy in experimental diabetes: part I. streptozotocin-induced rat models. Neurourology and Urodynamics 37 (2018) 1212-1219.

[8] J.H. Ellenbroek, E. Arioglu Inan, and M.C. Michel, A systematic review of urinary bladder hypertrophy in experimental diabetes: Part 2. Comparison of animal models and functional consequences. Neurourology and Urodynamics 37 (2018) 2346-2360.

[9] E.M. Kudlacz, A.L. Chun, K.A. Skau, M.C. Gerald, and L.J. Wallace, Diabetes and diuretic-induced alterations in function of rat urinary bladder. Diabetes 37 (1988) 949-955.

[10] B. Eika, R.M. Levin, and P.A. Longhurst, Comparison of urinary bladder function in rats with hereditary diabetes insipidus, streptozotocin-induced diabetes mellitus, and nondiabetic osmotic diuresis. Journal of Urology 151 (1994) 496-502.

[11] Y. Fukumoto, M. Yoshida, R.M. Weiss, and J. Latifpour, Reversability of diabetes- and diuresis-induced alterations in rat bladder dome muscarinic receptors. Diabetes 43 (1994) 819-826.

[12] T.L.J. Tammela, J.A.K. Briscoe, R.M. Levin, and P.A. Longhurst, Factors underlying the increased sensitivity to field stimulation of urinary bladder strips from streptozotocin-induced diabetic rats. British Journal of Pharmacology 113 (1994) 195-203.

[13] T.L.J. Tammela, R.E. Leggett, R.M. Levin, and P.A. Longhurst, Temporal changes in micturition and bladder contractility after sucrose diuresis and streptozotocin-induced diabetes mellitus in rats. Journal of Urology 153 (1995) 2014-2021.

[14] G. Liu, and F. Daneshgari, Alterations in neurogenically mediated contractile responses of urinary bladder in rats with diabetes. American Journal of Physiology 288 (2005) F1220-F1226.

[15] N. Xiao, Z. Wang, Y. Huang, F. Daneshgari, and G. Liu, Roles of polyuria and hyperglycemia in bladder dysfunction in diabetes. The Journal of Urology 189 (2013) 1130-1136.

[16] Z.E. Yesilyurt, B.R. Erdogan, I. Karaomerlioglu, A.E. Müderrisoglu, M.C. Michel, and E. Arioglu Inan, Urinary bladder weight and function in a rat model of mild hyperglycemia and its treatment with dapagliflozin. Frontiers in Pharmacology 10 (2019) 911.

[17] C. Kilkenny, W. Browne, I.C. Cuthill, M. Emerson, and D.G. Altman, Animal research: reporting in vivo experiments: The ARRIVE guidelines. British Journal of Pharmacology 160 (2010) 1577-1579.

[18] N. Percie du Sert, V. Hurst, A. Ahluwalia, S. Alam, M.T. Avey, M. Baker, W.J. Browne, A. Clark, I.C. Cuthill, U. Dirnagl, M. Emerson, P. Garner, S.T. Holgate, D.W. Howells, N.A. Karp, S.E. Lazic, K. Lidster, C.J. MacCallum, M. Macleod, E.J. Pearl, O.H. Petersen, F. Rawle, P. Reynolds, K. Rooney, E.S. Sena, S.D. Silberberg, T. Steckler, and H. Würbel, The ARRIVE guidelines 2.0: updated guidelines for reporting animal research. British Journal of Pharmacology 177 (2020) 3617-3624.

[19] J. Vollert, E. Schenker, M. Macleod, A. Bespalov, H. Wuerbel, M. Michel, U. Dirnagl, H. Potschka, A.-M. Waldron, K. Wever, T. Steckler, T. van de Casteele, B. Altevogt, A. Sil, and A.S.C. Rice, Systematic review of guidelines for internal validity in the design, conduct and analysis of preclinical biomedical experiments involving laboratory animals. BMJ Open Science 4 (2020) e100046.

[20] M.C. Michel, T.J. Murphy, and H.J. Motulsky, New author guidelines for displaying data and reporting data analysis and statistical methods in experimental biology. Molecular Pharmacology 97 (2020) 49-60.

[21] M.S. Islam, Animal models of diabetic neuropathy: progress since 1960s. J Diabetes Res 2013 (2013) 149452-149452.

[22] S. Lenzen, Animal models of human type 1 diabetes for evaluating combination therapies and successful translation to the patient with type 1 diabetes. Diabetes/Metabolism Research and Reviews 33 (2017) e2915-n/a.

[23] E. Ahlqvist, R.B. Prasad, and L. Groop, Subtypes of type 2 diabetes determined from clinical parameters. Diabetes 69 (2020) 2086-2093.

[24] Z.E. Yesilyurt, B.M. Ertürk, B.R. Erdogan, E. Arioglu-Inan, and M.C. Michel, Effects of the sodium-glucose transporter 2 inhibitor empagliflozin on bladder size, contraction and relaxation in a rat model of type 1 diabetes. Neurourol. Urodyn. in press (2021).

[25] M.C. Michel, and E. Arioglu-Inan, Function and morphology of the urinary bladder after denervation. American Journal of Physiology: Regulatory, Integrative and Comparative Physiology 320 (2021) R833-R834.

[26] M.C. Michel, E. Mayoux, and V. Vallon, A comprehensive review of the pharmacodynamics of the SGLT2 inhibitor empagliflozin in animals and humans. Naunyn-Schmiedebergs Archives of Pharmacology 388 (2015) 801-816.

[27] J.M. Ikle, and A.L. Gloyn, 100 years of insulin: A brief history of diabetes genetics: insights for pancreatic beta-cell development and function. J Endocrinol 250 (2021) R23-r35.

[28] I.-H. Chen, J.-T. Cheng, and Y.-C. Tong, Metabolic syndrome induced bladder cannabinoid receptor changes in the fructose-fed rats. LUTS: Lower Urinary Tract Symptoms 10 (2018) 198-203.

# Data Availability Statement

The datasets generated for this study are available from the authors upon reasonable request.
